# Pre‐silking water deficit in maize induced kernel loss through impaired silk growth and ovary carbohydrate dynamics

**DOI:** 10.1002/pei3.10141

**Published:** 2024-04-06

**Authors:** Yebei Li, Shoubing Huang, Qingfeng Meng, Zongxin Li, Felix B. Fritschi, Pu Wang

**Affiliations:** ^1^ College of Agronomy and Biotechnology China Agricultural University Beijing China; ^2^ Division of Plant Science and Technology University of Missouri Columbia Missouri USA; ^3^ Shandong Academy of Agricultural Science Jinan China

**Keywords:** kernel number reduction, maize, reproductive organ, silk extrusion, sugar status, water deficit

## Abstract

Both carbon limitation and developmentally driven kernel failure occur in the apical region of maize (*Zea mays* L.) ears. Failed kernel development in the basal and middle regions of the ear often is neglected because their spaces usually are occupied by adjacent ovaries at harvest. We tested the spatial distribution of kernel losses and potential underlying reasons, from perspectives of silk elongation and carbohydrate dynamics, when maize experienced water deficit during silk elongation. Kernel loss was distributed along the length of the ear regardless of water availability, with the highest kernel set in the middle region and a gradual reduction toward the apical and basal ends. Water deficit limited silk elongation in a manner inverse to the temporal pattern of silk initiation, more strongly in the apical and basal regions of the ear than in the middle region. The limited recovery of silk elongation, especially at the apical and basal regions following rescue irrigation was probably due to water potentials below the threshold for elongation and lower growth rates of the associated ovaries. While sugar concentrations increased or did not respond to water deficit in ovaries and silks, the calculated sugar flux into the developing ovaries was impaired and diverged among ovaries at different positions under water deficit. Water deficit resulted in 58% kernel loss, 68% of which was attributable to arrested silks within husks caused by lower water potentials and 32% to ovaries with emerged silks possibly due to impaired carbohydrate metabolism.

## Graphical abstract

Pre‐silking water stress (WS) exacerbated kernel loss throughout the whole ear, with the increased arrested silks contributing more than ovary failure (68% vs. 32%). WS had different extents of limitation for silks at different positions, and the severity of impact was opposite to the temporal pattern of silk initiation.

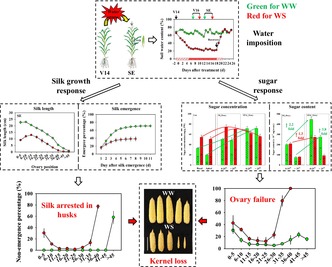

## 1 INTRODUCTION

In maize (*Zea mays* L.), a disparity between the number of established florets and the number of kernels at harvest is still common, especially under abiotic stress (DeBruin et al., 2018; Oury, Tardieu, et al., 2016; Shen et al., 2020; Wang et al., 2021). In general, the response to water deficit among yield components is more plastic for kernel number per ear compared to ears per area and weight per kernel (Borrás & Vitantonio‐Mazzini, 2018). Consequently, many studies have focused on the underlying mechanisms for water stress (WS)‐induced maize kernel loss using both field (Asch et al., 2001; Otegui et al., 1995; Shen et al., 2019; Westgate & Boyer, 1986) and controlled environment studies (McLaughlin & Boyer, 2004a, 2004b; Oury, Tardieu, et al., 2016; Oury, Caldeira, et al., 2016; Zinselmeier et al., 1999). It is well established that water deficit can cause decreased kernel numbers as a result of extended anthesis‐silking intervals (ASIs) and by inducing kernel abortion (Bolaños & Edmeades, 1990, 1993, 1996; McLaughlin & Boyer, 2004b; Zinselmeier et al., 1999). Further, for maize exposed to a water deficit stress initiated at tassel emergence, Oury, Tardieu, et al. (2016) observed increased rates of abortion of young ovaries with silks that did not emerge at least 2 days before water deficit stress‐induced silk growth arrest, thus linking shorter duration of silk elongation after silk emergence (SE) to increased ovary abortion. These findings indicate the importance of silk elongation and extrusion for the proper development of ovaries and successful fertilization. Thus, it is of interest to clarify the causal link between silk growth and kernel losses, especially under early water deficit.

In the field, faster and more synchronous silk extrusion is beneficial for successful kernel establishment and kernel number per ear at harvest (Borrás & Vitantonio‐Mazzini, 2018; DeBruin et al., 2018; Rossini et al., 2020). Silk appearance follows a sequential order with silks in middle and basal regions of the ear emerging first while apically positioned silks lag behind by 3–4 days (Cárcova et al., 2003). This results in a pollination window of several days among ovaries at different positions along the ear. In turn, early pollinated ovaries may interfere with the survival and development of the later pollinated ones as a result of signaling and the discrepancy in sink strength considering ovary growth rate and/or sugar status, particularly under water deficit conditions (Cárcova & Otegui, 2001, 2007; Setter & Parra, 2010; Shen et al., 2018). Aggravated by water deficit conditions, late‐developing silks may cease to grow within husks or late emergence may be associated with a short interval until silk growth arrest and impaired ability of pollen tube growth, possibly as a result of silk tissue stiffening (Bassetti & Westgate,1993c; Fuad‐Hassan et al., 2008; Oury, Tardieu, et al., 2016; Turc & Tardieu, 2018). Thus, establishment of apical kernels generally is more tenuous than that of middle and basal ones, which often is manifested as ear tip‐barrenness. Although failure of kernel set and development at the ear tip can be observed readily, kernel losses also occur throughout the remainder of the ear, but are more easily overlooked because they are less obvious at harvest (Gustin et al., 2018). Given that silk initiation is coupled with the crucial period of ovary expansion, which begins about 2 weeks prior to tassel emergence (Fuad‐Hassan et al., 2008), we hypothesized that water deficit stress during silk elongation will alter developmental processes of reproductive structures in a manner that will impact the spatial distribution of kernel losses within an ear.

Carbon starvation may lead to kernel abortion in maize when water deficit stress occurs for 6 days after silking with hand pollination on the fifth day (McLaughlin & Boyer, 2004a). This is consistent with observations by Boyle et al. (1991) and Zinselmeier et al. (1995, 1999) that sucrose infusion during a 6‐day postsilking water deficit can limit the extent of kernel abortion. Nonetheless, although Schussler and Westgate (1995) documented increased sugar concentrations in ovaries under water deficit stress imposed during SE until 2 days after pollination, about 40% kernel loss still occurred. Oury, Tardieu, et al. (2016) also observed a 29%–77% loss in grain number when maize suffered mild to severe water deficit stress from tasseling to 6 days after SE. In their study, developmental arrest of silks rather than carbon starvation was the trigger in more than 90% of water‐stress induced ovary abortions. While WS resulted in sugar concentrations that were maintained or elevated in both ovaries and silks before as well as after the occurrence of ovary abortion, the amount of sugar per organ was at least maintained only before silking but not days after SE as it closely followed the change of growth of reproductive organs instead of the switch of abortion (Oury, Caldeira, et al., 2016). Thus, the timing as well as the severity of the water deficit stress appear to influence the reasons underlying ovary/kernel abortion. Ovary abortion is primarily due to a change of sequential development of reproductive organs when stress occurs before 3 days after SE (Turc & Tardieu, 2018), and carbohydrate limitations are supposed to be the main cause of kernel abortion when stress occurs during 2 weeks after silking (Boyer & Westgate, 2004). Previously, Fuad‐Hassan et al. (2008) conducted a detailed examination of silk expansion and its cell division and cell elongation components in response to water deficit stress. They observed that the kinematic pattern of whole silk development was largely conserved, but that water deficit stress increased the duration of phase 3 (end of cell division until cessation of cell growth in the silk apex), which was closely linked with the ASI. However, the direct causality between the impacts of water deficit stress on silk elongation and the destiny of the florets still needs further exploration. Concurrent assessment of ovary and silk growth together with carbohydrate dynamics may enhance our understanding of the spatial pattern of ovary development and fate in response to water deficit imposed during silk elongation.

Here, we conducted greenhouse experiments to quantify the impact of water deficit imposed during silk elongation on the pattern of kernel loss along the length of the ear, and to further explore the reasons underpinning the kernel loss pattern from the perspectives of silk growth and sugar status of reproductive organs. To this end, we sampled ears at different timepoints relative to WS imposition and characterized the spatial distribution of ovaries with and without emerged silks, ovaries that failed to develop into kernels even with emerged silks, and established kernels along the length of the ear. Additionally, we measured silk lengths, ovary fresh weights, and ovary and silk sugar concentrations and contents in different regions of the ear. In turn, we explored how these characteristics were associated with the fate of ovaries at different positions along the length of the ear.

## 2 MATERIALS AND METHODS

### 2.1 Plant material and growth condition

Two pot experiments were conducted in February through April (Exp I) and in August through October (ExpII) 2018 in a greenhouse at the University of Missouri, Columbia, MO, USA. The maize inbred line B73 was grown in 18.9‐L pots filled with 3.5 kg (Exp I) or 4.0 kg (Exp II) of well‐mixed potting medium (general‐purpose PRO‐MIX BX). One day before planting, each pot was saturated with Hoagland nutrient solution [6 mM Ca(NO_3_)_2_, 4 mM KNO_3_, 2 mM KH_2_PO_4_, 2 mM MgSO_4_, 50 μM Fe‐Citrate, 25 μM H_3_BO_3_, 10 μM MnSO_4_, 2 μM ZnSO_4_, 0.5 μM CuSO_4_, and 0.5 μM H_2_MoO_4_ with pH at 6.0–7.0] (McLaughlin & Boyer, 2004a, 2004b). Seeds were pregerminated in water overnight, and three seeds per pot were planted at a depth of 3–4 cm in 96 pots for Exp I and 108 pots for Exp II. At the 3‐ or 4‐leaf stage, seedlings were thinned to one per pot. Photoperiod in the greenhouse was set to 16 h/8 h (day/night) by supplementing natural light with metal halide lamps positioned above the plant canopy, as described in Peng et al. (2013). The temperature was set at 26.7 ± 3°C, while the actual temperature was recorded every 15 min through HOBO Pro Series data loggers (Onset Computer Corporation, USA). Pots were arranged in a randomized block design with four replications, each consisting of two rows of 24 plants in both experiments, plus one extra single row of 12 plants in Exp II. Within each replication, half of the plants in each row were destined for the well‐watered (WW) and the other half for the WS treatment. The 12 extra plants in Exp II were evenly divided into WW and WS plants at the onset of the WS treatment, and were assigned into replicates as spaces opened up when destructive samplings were conducted. Within a row, plants were spaced 0.3 m apart and the two rows of plants in each replication also were separated by 0.3 m. To provide easy access to each plant, replications were spaced 1.1 m apart. To minimize differences caused by microenvironment conditions in the greenhouse, pots were rearranged two times before the initiation of WS treatments. Hand pollination was conducted daily from the first day of SE until SE stopped.

### 2.2 Water regimes

Prior to WS treatment imposition, plants were supplied with nutrient solution (described above) as needed to maintain WW conditions. The content of KNO_3_ in the nutrient solution was increased to 12 mM starting at the 6‐leaf stage. Once plants reached the 14‐leaf stage (V14), all pots were saturated with deionized water just before treatment initiation, half of the plants were assigned to the WW treatment and the other half to the WS treatment. The weight of each pot was recorded daily to determine soil water content and irrigation amount for each pot. WW plants were irrigated daily to maintain soil moisture at 60%–80% of water holding capacity (gravity drained overnight following saturation), while weights in the WS treatment were allowed to decrease to 30% of water holding capacity and were then maintained at this level by daily addition of small amounts of water based on pot weight until 3 days after SE. At that point, water additions were increased to allow soil water content to gradually increase over 3 days, after which all plants were maintained WW until the final sampling.

### 2.3 Leaf water potential and photosynthesis measurements

Leaf water potentials were determined with a pressure chamber (Model 1000, PMS Instrument Company, USA). Selected leaves were clipped, immediately inserted into the chamber, slowly pressurized, and water potential values were recorded when water first appeared at the cut leaf surface. Predawn leaf water potentials were determined every 2–3 days, whereas midday (11:30–13:30) leaf water potentials were determined once pot weights reached 30% of water content in the WS treatment, 2 days prior to rewatering, and 3 days after rewatering in Exp I, and prior to stress imposition, on the fourth day of water withholding, 1 day prior to rewatering, and 4 days after rewatering in Exp II. At each time point, the tips (10–20 cm) of one to three leaves from different plants in each replicate of both treatments were used for water potential measurements. Predawn measurements were conducted on green leaves from lower stem nodes, while ear leaves or the topmost fully expanded leaves were measured at midday.

Net photosynthesis was measured every 2–3 days starting 1 day before WS treatment initiation until five (Exp I) or seven (Exp II) days after rewatering, using an open gas‐exchange system (LI 6400, Li‐Cor Inc., Lincoln, NB, USA) set to 1500 μmol m^−2^ s^−1^ photosynthetic photon flux density, 400 μmol mol^−1^ CO_2_, 500 mmol s^−1^ flow rate, and 25°C leaf temperature. Measurements were conducted on the middle third of the ear leaves or, in instances where ear leaves were not fully expanded yet, the topmost fully expanded leaves of at least four plants per treatment in each replication.

### 2.4 Flowering time and SE dynamic

Plant growth and development were followed throughout the experiment to record key developmental stages and to time the initiation of water treatment imposition as well as measurements and sample collections. Dates of tasseling, pollen shedding, and silking were recorded based on 22 or more plants for each water treatment. Silk extrusion dynamics were assessed by counting emerged silks at the end of the day starting with the first day of SE. After counting, newly extruded silks were cut with a scissor 1 cm above the husk each day, and only the freshly extruded silks with bifurcated tips were counted the following day until silk extrusion ceased.

### 2.5 Ear and silk lengths, fractionations, and ovary fresh weights

Ear lengths were measured at the 14‐leaf stage (V14), the 16‐leaf stage (V16), on the first day of SE, and 6 days after silking (SE+6), on 6–14 plants per treatment at each timepoint. At V14, ears were removed from two plants per treatment from three replications, husks were removed to expose the ear, and ear length was measured. At V16, ears were removed from one plant per treatment from three replications for tissue water potential measurements using a thermocouple psychrometer, from one plant per treatment from three replications for observation of florets under the microscope, and from two plants per treatment from all replications for sugar measurements. The lengths of all ears at V16 were measured and the top 1/3 of each ear (including silks) was categorized as apical tissue, and the remaining 2/3 of each ear were categorized as basal tissue. The apical and basal tissues of the ears destined for soluble sugar analyses were packaged separately in aluminum foil, immersed in liquid N, and stored at −60°C until they were used for quantification of sugars. At SE and SE+6, ears from two plants per treatment from three replications were gently shucked, lengths measured, and tissues were divided into 2 (Exp I) or 3 (Exp II) groups based on kernel position within a row (ring position) on the ear. Specifically, silks and ovaries were detached from the ears and divided into basal (0–25th kernel position) and apical tissues (>25th kernel position) in Exp I, and into basal (0–10th kernel position), middle (11–25th kernel position), and apical tissues (>25th kernel position) in Exp II. Six rows of tissues from each position were immediately detached from all the ears, packaged in aluminum foil, flash frozen in liquid N, and stored at −60°C until quantification of soluble sugars as described below. Half of them (one plant per treatment from three replications) were immediately used for tissue water potential measurements using a thermocouple psychrometer. After removal of the six rows, the ears from three plants per treatment in Exp I, and all ears from Exp II (6 plants at SE, 5 WW plants and 6 WS plants at SE+6) were placed in a refrigerator at 4°C, and silk lengths and ovary fresh weights were measured within a few days. Six rows of silks were detached from these ears, and the lengths of silks were measured one by one. Silk lengths at SE were measured from the insertion point to the silk tip, grouped every five rings in Exp I and for every ring in Exp II. The distance between silk tips and husk tips was recorded at SE+6 for every ring in both experiments, with distances recorded as 0 for emerged silks. Ovaries from six rows of each ear were detached and weighed after removal of silks. Individual ovary fresh weights were computed based on the weight of grouped ovaries divided by the number of ovaries per group, with groups corresponding to the different regions of the ear based on the aforementioned ear sections.

### 2.6 Occupancy of different types of ovaries

At the end of each experiment, ears were detached from five to eight plants per treatment to characterize the fate of the florets. All ears within an experiment were harvested on the same day which, because of the differences in development between the treatments, corresponded to 4–7 days for Exp I and 6–12 days for Exp II after the last manual pollination. All ears were in the initial stages of kernel development which was beneficial to easily recognize different types of ovary fates. The percentages of different ovary types were determined as follows: After cutting the emerged silks with a razor blade, husks were gently removed to expose the ear. The silks with cut cross‐sections were counted and recorded as emerged silk number, while those tightly attached and with bifurcated tips were categorized as non‐emerged silks. All visible kernels and ovaries were counted and recorded as part of the total floret number, and swelled kernels were counted as established and recorded as kernel number. The percentages of each type of ovary in the different regions of the ear were computed using every five rings of ovaries as a group in Exp I and each ring as a group in Exp II. Specifically, the percentage of kernel setting was calculated by dividing kernel number by the total floret number, and the percentage of non‐emerged silks by dividing the number of non‐emerged silks by the total floret number. The percentages of failed ovaries were calculated using the number of emerged silks minus the kernel number as the numerator and the number of emerged silks as the denominator.

Kernel losses were separated into three different categories, including WS induced decrease in differentiated florets (Loss 1), non‐emerged silks in response to WS (Loss 2), and failed kernel development despite silk emergence under WS (Loss 3), all expressed on the basis of the potential maximal number of kernels under WW conditions that failed to set when plants were grown under WS conditions. The percentages contributed by each of these categories of kernel losses under water deficit were calculated using the following equations:

$$\begin{array}{c} \text{Loss}\;1\;\left(\%\right)=\\ 100\times \frac{\text{Total floret number of}\;\mathrm{WW}\;\text{plants}-\text{Total floret number of}\;\mathrm{WS}\;\text{plants}}{\text{Total floret number of}\;\mathrm{WW}\;\text{plants}-\text{Kernel number of}\;\mathrm{WS}\;\text{plants}} \end{array}$$

$$\begin{array}{c} \text{Loss}\;2\;\left(\%\right)=\\ 100\times \frac{\text{Total floret number of}\;\mathrm{WS}\;\text{plants}-\text{Emerged silk number of}\;\mathrm{WS}\;\text{plants}}{\text{Total floret number of}\;\mathrm{WW}\;\text{plants}-\text{Kernel number of}\;\mathrm{WS}\;\text{plants}} \end{array}$$

$$\begin{array}{c} \text{Loss}\;3\;\left(\%\right)=\\ 100\times \frac{\text{Emerged silk number of}\;\mathrm{WS}\;\text{plants}-\text{Kernel number of}\;\mathrm{WS}\;\text{plants}}{\text{Total floret number of}\;\mathrm{WW}\;\text{plants}-\text{Kernel number of}\;\mathrm{WS}\;\text{plants}} \end{array}$$

### 2.7 Soluble sugar concentrations and contents

Soluble sugars, including glucose, fructose, and sucrose, were quantified according to Ning et al. (2018). Briefly, stored ovaries and silks were ground with the pestle and mortar in liquid N and then transferred into tubes. Samples of 20 mg of fresh powder were extracted in 80% (v/v) ethanol in a water bath at 80°C for 15 min, followed by centrifugation for 5 min. The extraction was repeated three times for each sample and all three supernatants were pooled and the final volume was adjusted to 2 mL. The liquid was filtered through 0.45 μm nylon syringe filters (Thermo Scientific, USA), and 20 μL was used for the separation of sugars by HPLC (Shimadzu Corp., Kyoto, Japan). A Luna 5 μm NH_2_ 100 Å, LC column (size: 250 × 4.6 mm) (Phenomenex Inc.) was used with a mobile phase of 80:100 (v/v) = acetonitrile: water for sugar separation. The flow rate of the mobile phase was set at 0.75 mL min^−1^ and the temperature at 30°C. A refractive index detector (Model RID‐10A) was used for glucose, fructose, and sucrose detection. The “LabSolutions” (Shimadzu Corp.) software was used for post‐run data analysis.

### 2.8 Statistical analyses

Analysis of variance (ANOVA) was conducted to test the effect of water treatments on leaf water potential, net photosynthesis, and ear length, and the effect of water treatments and ovary positions or sampling timepoints on ovary fresh weights, sugar concentrations, and sugar contents. A liner mixed model was runned with lm4 package in R with the tissue positions and treatments or timepoints as fixed effects and replications and plants as random effects. Fisher's least significant difference was used for the separation of the mean values with significant differences at *p* = .05 level. Student's *t*‐test was used to test for differences between WW and WS treatments in flowering time, and the indicators collected at harvest, including total floret number, emerged silks, final kernel number, and ear lengths, with a 95% confidence interval. Statistical analyses were perfomed in R version 3.6.1 (https://www.r‐project.org), and figures and linear regressions were generated in SigmaPlot 12.5 (Systat Software, Inc.).

## 3 RESULTS

### 3.1 Impact of water treatments on plant water status and net photosynthesis

**FIGURE 1:**
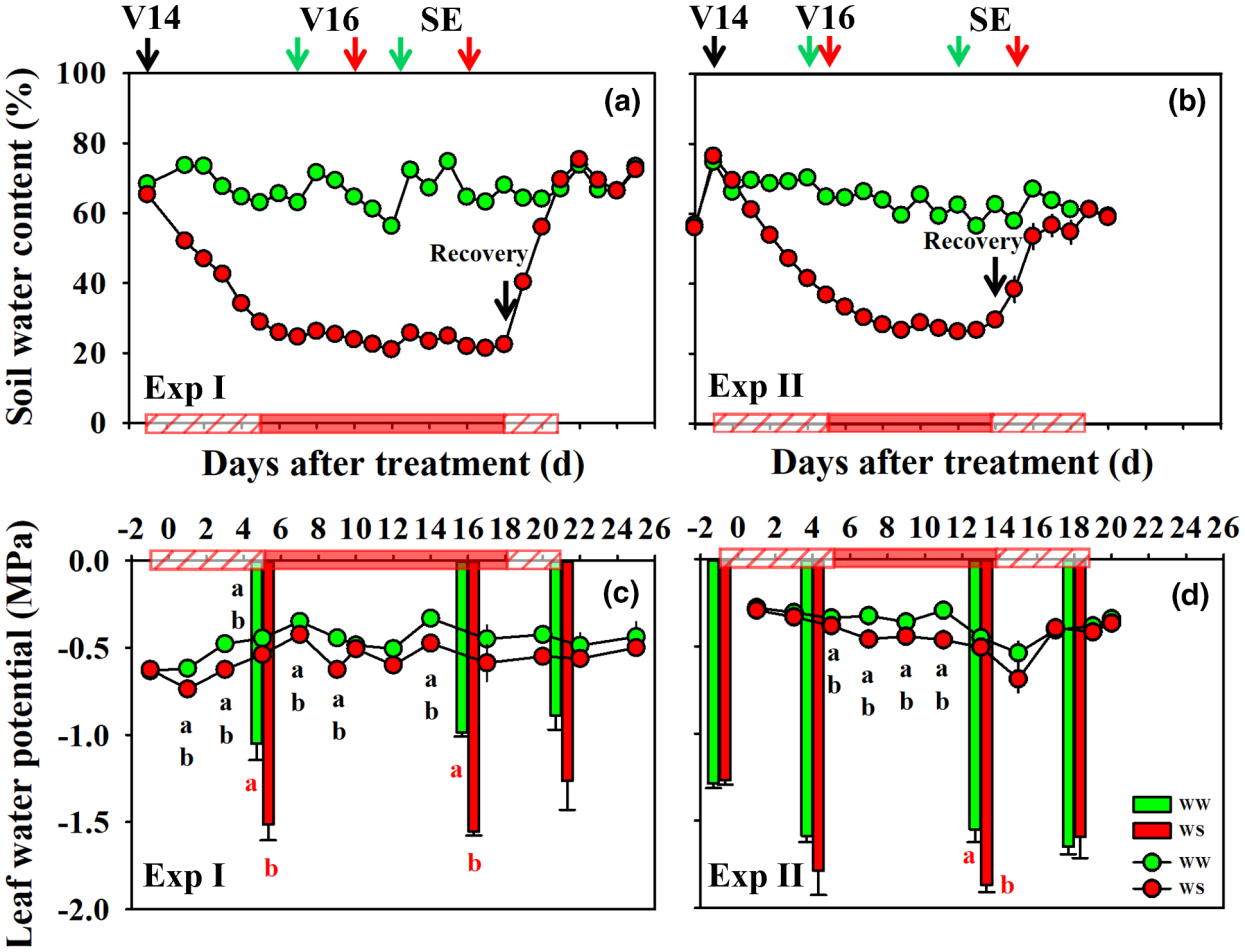
The daily dynamic of soil water content in Experiment I (Exp I, a) and Experiment II (Exp II, b), and pre‐dawn (lines) and midday (bars) leaf water potentials in Exp I (c) and Exp II (d) under well‐watered (WW, green) and water‐stressed (WS, red) conditions. Error bars stand for standard errors. Black arrows above panels a and b point out the initiation of water treatment at the 14‐leaf stage (V14), and green and red arrows show the critical stages of WW and WS plants, respectively. V16, 16‐leaf stage; SE, the first day of silk emergence. The black arrows inside panels a and b represent the timepoint of water recovery for WS plants; Boxes filled with red diagonal lines on *X*‐axis represent soil moisture of WS plants during the decreasing process after irrigation withholding or increasing process after water recovery, while boxes filled with red color represent WS plants at the target soil moisture level. Different letters in panels c and d mean significant differences in pre‐dawn (black letters) and midday (red letters) leaf water potentials between treatments at the *p* < .05 level, and error bars stand for SEs.

Cessation of watering in the WS treatments at V14 caused soil water content to fall to approximately 30% within 5–6 days in the two experiments (Figure 1a,b). Regular watering in small increments based on daily pot weight assessments, resulted in a relatively consistent level of soil water content in the WS treatments until WS was terminated by rewatering to full water holding capacity. Pre‐dawn and mid‐day water potential measurements indicated successful imposition of water deficit stress during the desired developmental stages, and recovery of the plant water status after rewatering (Figure 1c,d). Specifically, during the time window when the WS treatments were maintained at 30% soil water content, average pre‐dawn leaf water potentials were 0.11 MPa (−0.54 vs. −0.43 MPa) and 0.10 MPa (−0.45 vs. − 0.35 MPa) lower than those in WW treatments in the Exp I and Exp II, respectively. Mid‐day leaf water potentials measured 16 and 13 days post initiation of the WS treatments averaged −1.56 and − 1.87 MPa and were 0.57 and 0.32 MPa lower than those in WW treatments in Exp I and Exp II, respectively.

Net photosynthetic rates (*P*_n_) decreased rapidly in response to water deficit stress imposition, and recovered considerably, but not completely, after re‐watering (Figure S1a,b). On average, for the period when soil water content was maintained at 30%, *P*_n_ of WS plants was 49% and 44% that of WW plants in Exp I and Exp II, respectively. Following rewatering, *P*_n_ of WS plants partially recovered, reaching 89% (Exp I) and 71% (Exp II) that of WW plants within 2 days of full irrigation.

### 3.2 Non‐emerged silks accounted for a large proportion of water deficit‐induced kernel losses

**FIGURE 2:**
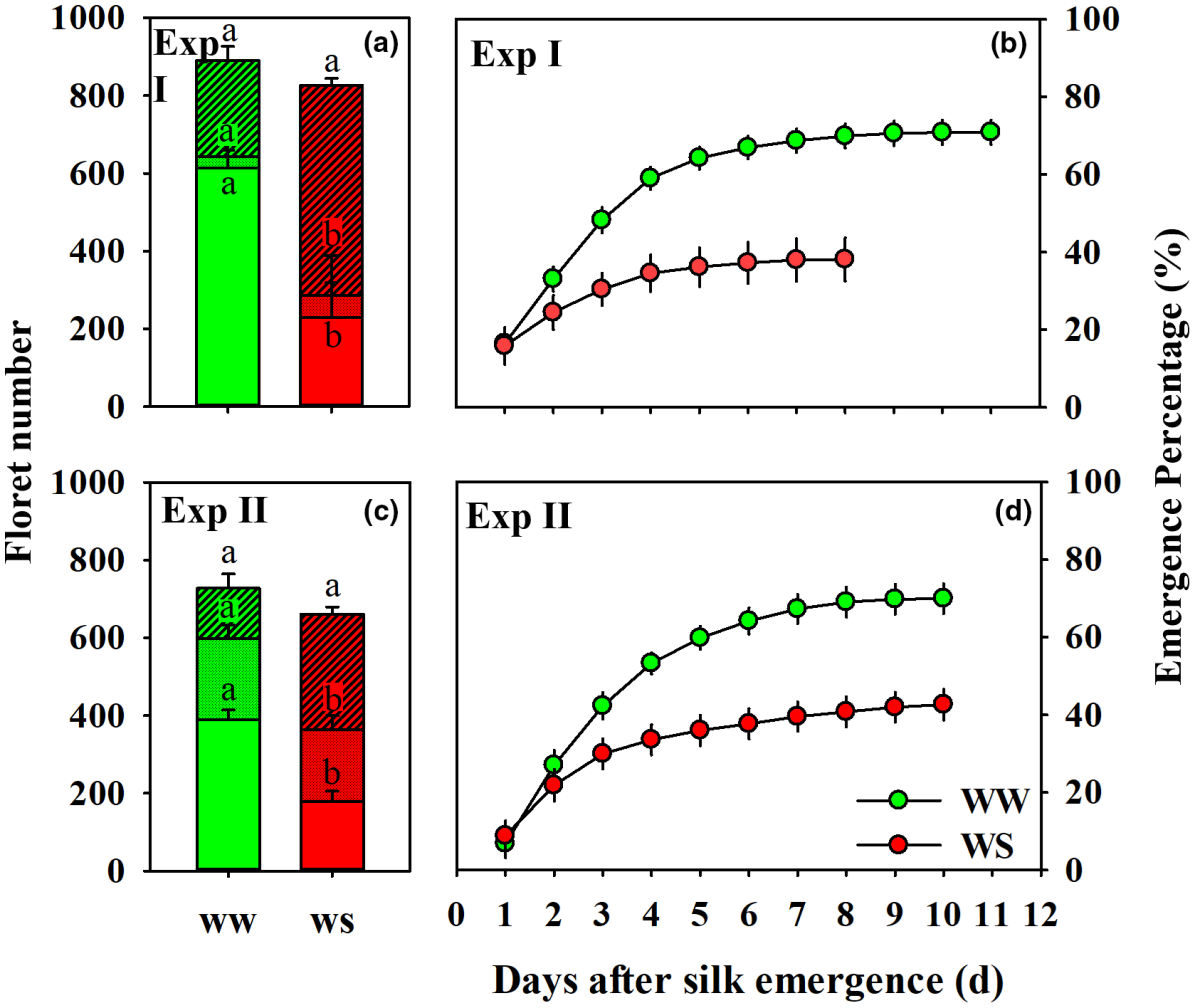
Kernel number (blank filled bars), emerged silk number (dot filled bars), total floret number (slash filled bars) of well‐watered (green, WW) and water‐stressed (red, WS) plants in Experiment I (Exp I, a) and Experiment II (Exp II, c), and the dynamic of accumulated silk emergence percentages of WW and WS plants in Exp I (b) and Exp II (d). Error bars stand for SE. Different letters in panels a and c mean significant differences between treatments at the *p* < .05 level.

Although the total number of visible florets per ear was not different between WW and WS treatments, the WS treatment dramatically reduced the number of kernels per ear (230 in Exp I and 179 in Exp II) compared to the WW treatment (614 in Exp I and 390 in Exp II; Figure 2a,c). Consistent with the severely reduced number of kernels, WS reduced both the number of silks that emerged per day (Exp I and Exp II) as well as the duration of silk emergence (Exp I; Figure 2b,d). Consequently, the total number of emerged silks in the WS treatment was only 42% (Exp I) and 61% of the 835 (Exp I) and 597 (Exp II) silks that emerged under WW conditions, respectively. In this study, compared to WW conditions, WS delayed the time to tasseling, pollen shedding, and silking by 1.8 d, 1.0 d, and 2.9 d in Exp I and by 1.1 d, 0.9 d, and 2.5 d in Exp II. Thus, WS prolonged the ASI by 1.9 d in Exp I and by 1.6 d in Exp II (Figure S2), respectively. Since all harvested ears were pollinated by hand to supply sufficient fresh pollen grains, failed ovaries were not a consequence of a lack of pollen grains, which could result from the prolonged ASI. To elucidate the contributions of other processes to the extent of kernel number reductions (relative to potential number of kernels under WW conditions) observed in response to WS, we calculated the percentages of WS‐induced decreases in the number of differentiated florets (Loss 1), the number of non‐emerged silks in response to WS (Loss 2), and the number of kernels that failed to develop despite silk emergence under WS (Loss 3). These calculations revealed that contributions to the WS‐induced reduction in kernel set were 9.7% due to Loss 1, 81.9% due to Loss 2, and 8.4% due to Loss 3 in Exp I, whereas they amounted to 12.1% (Loss 1), 54.3% (Loss 2), and 33.6% (Loss 3) in Exp II. Thus, limited elongation of silks resulting in an increased number of non‐emerged silks was the main cause of the kernel number reduction in response to WS for approximately 2 weeks prior to silking.

### 3.3 WS impacted the extent but not the general pattern of distribution of established kernels, florets with non‐emerged silks, and failed ovaries along the length of the ear

**FIGURE 3:**
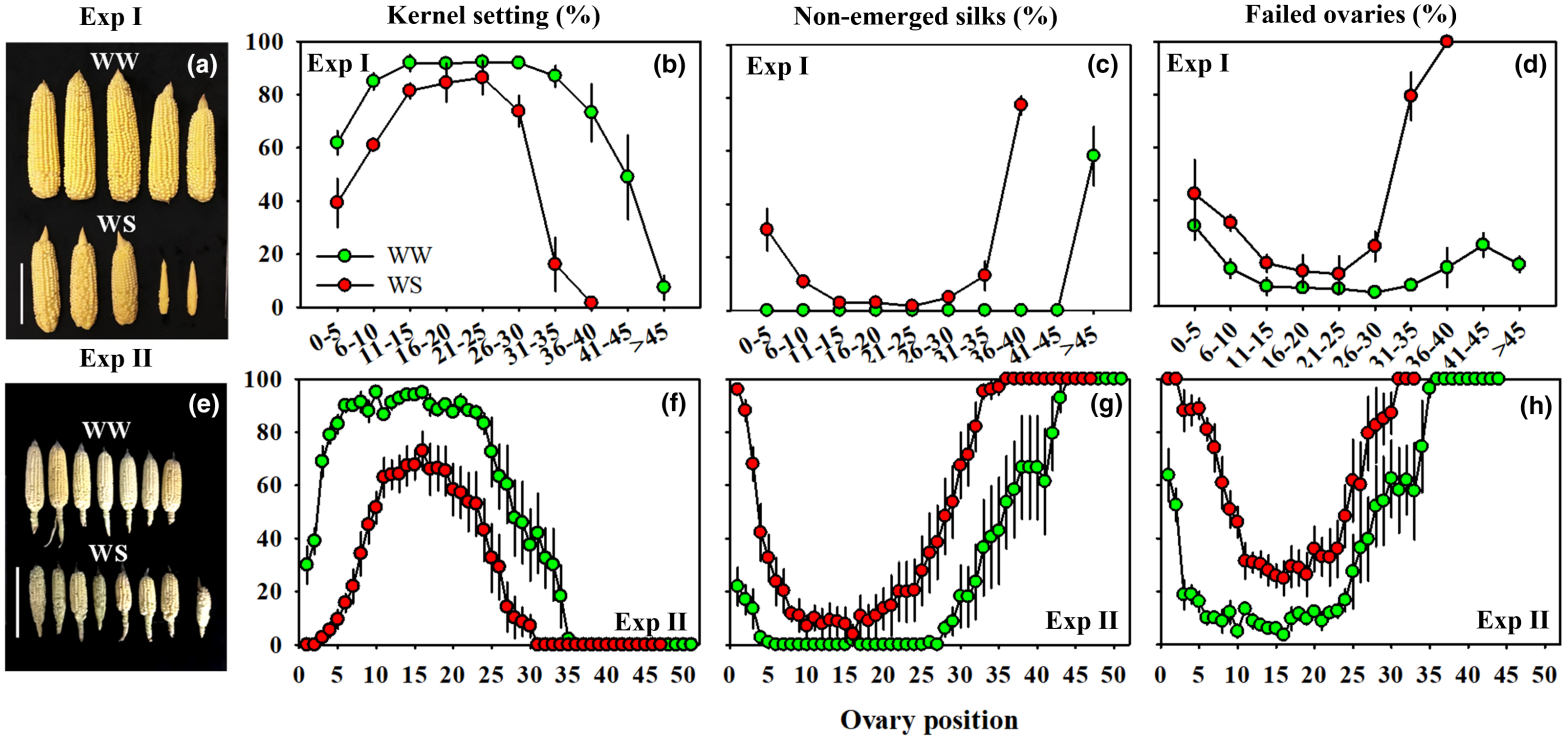
The well‐watered (WW) and water‐stressed (WS) ears harvested in Experiment I (Exp I, a) and Experiment II (Exp II, e), percentages of kernel setting (b for Exp I and f for Exp II), non‐emerged silks (c for Exp I and g for Exp II), and failed ovaries (d for Exp I and h for Exp II) along the whole length of the WW (green) and WS (red) ears. Error bars in panels b–d and f–h stand for SEs.

Similar patterns of kernel setting, non‐emerged silks, and failed ovaries were observed in both experiments (Figure 3). Kernel setting percentages were the greatest in the middle regions of the ear and gradually decreased toward the basal and apical ends of the ears in both WW and WS treatments (Figure 3b,f). However, WS plants consistently had lower kernel setting percentages across the entire length of the ear compared to WW plants, and the region in the middle of the ear with the greatest kernel set percentages was considerably narrower in the WS than the WW plants. Additionally, kernel set at the apical end was reduced in WS plants such that WW plants across the two experiments had approximately five more rings with successfully established kernels than WS plants.

The percentages of non‐emerged silks were greater in WS than WW treatments and showed a pattern that was opposite to those of kernel setting, with silk emergence limiting to a greater extent for basal and apical positions compared to middle positions on the ear (Figure 3c,g). Similarly, the percentages of ovaries that failed to develop into kernels (on the basis of ovaries with emerged silks) were greater in WS than WW treatments along the length of the ear, and were the lowest in the middle and increased toward the apical as well as the basal portions of the ears (Figure 3d,h).

### 3.4 WS limited the expansion of ears and silks, and reduced ovary mass

**FIGURE 4:**
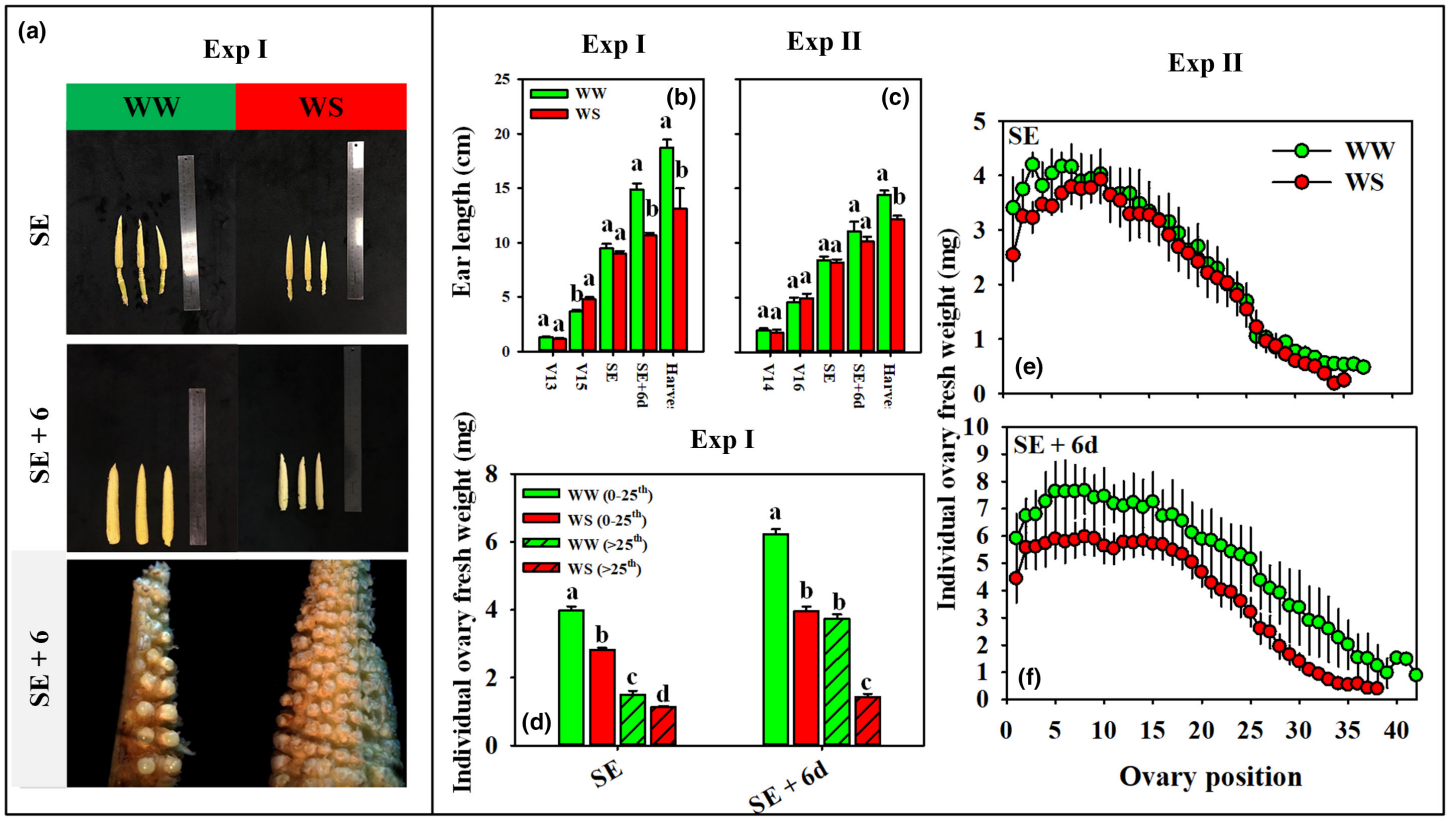
The morphology of well‐watered (WW) and water‐stressed (WS) ears at the first of silk emergence (SE) and 6 days after silk emergence (SE+6d) in Experiment I (Exp I, a), dynamic variation in lengths of WW (green) and WS (red) ears in Exp I (b) and Experiment II (Exp II, c), and fresh weights of basal (0–25th rings) and apical ovaries (>25th rings) of WW and WS ears at SE and SE+6d in Exp I (d), ovary fresh weight along the whole length of both WW and WS ears at SE (e) and SE+6d (f) in Exp II. Error bars in panels b–f stand for SEs. Different letters in panels b–d mean significant differences between treatments at the *p* < .05 level.

**FIGURE 5:**
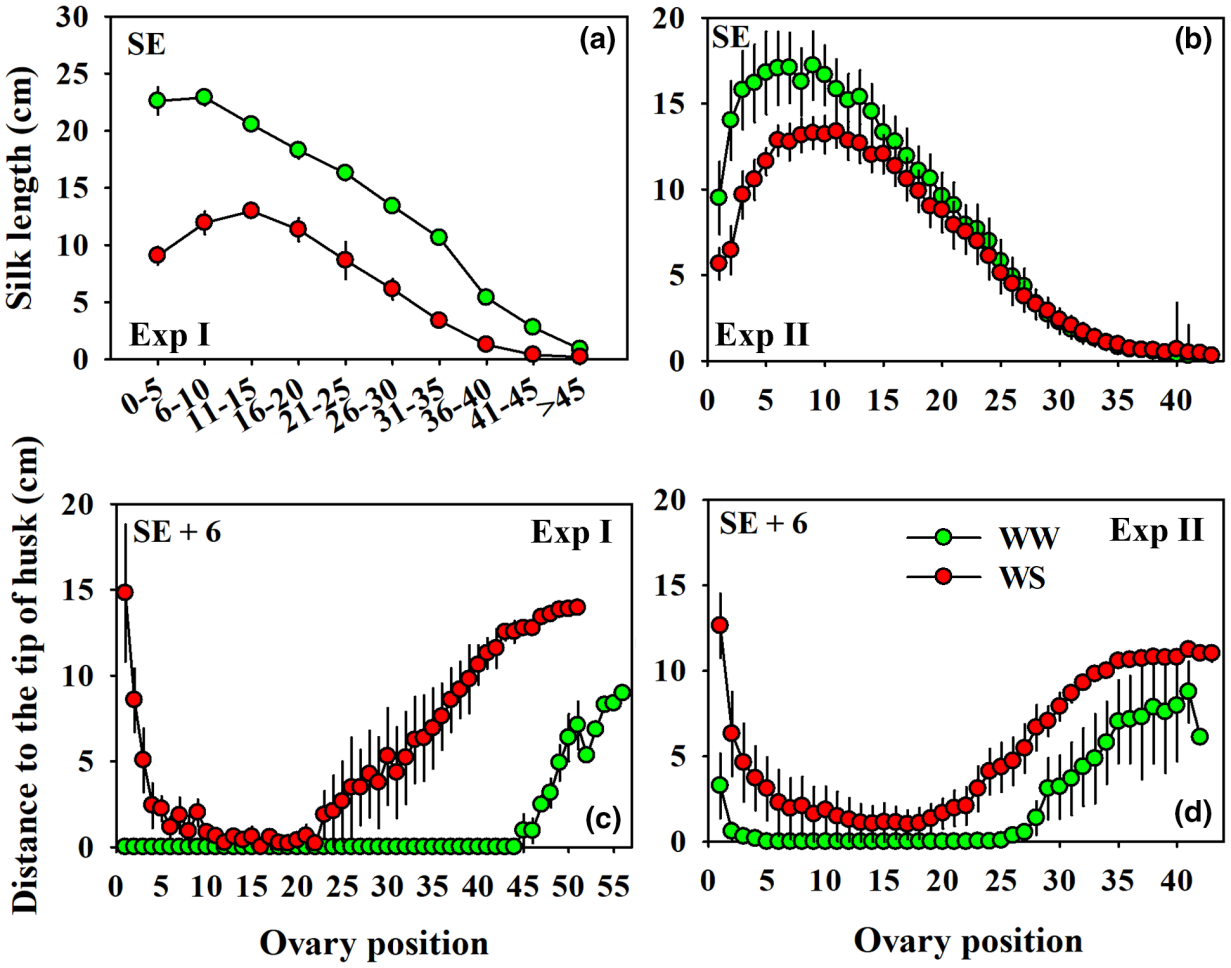
Silk lengths at the 1st of silk emergence (SE) in Experiment I (Exp I, a) and Experiment II (Exp II, b), and the distances between silk tips to husk tips at 6 days after silking (SE+6) in Exp I (c) and Exp II (d) across the entire length of both well‐watered (green, WW) and water‐stressed (red, WS) ears. Error bars stand for SEs.

Compared to the WW treatment, WS imposition starting at V14 resulted in shorter ears by the time plants reached SE+6d in Exp I and at the final sampling (SE+8–21d) in both experiments (Figure 4b,c). Ovary fresh weight (Figure 4d–f) and silk length and emergence (Figure 5) were more sensitive to water deficit imposition than ear length and were already detected at SE and became more pronounced by SE+6d. The WS treatment reduced ovary fresh weights in apical and basal halves of the ear at SE as well as SE+6d in Exp I (Figure 4d). In Exp II, ovary fresh weights in WW and WS treatments at SE were similar at most positions along the length of the ear, with small reductions observed in the WS treatment in the basal region of the ear (Figure 4e). As development progressed to SE+6d, the impact of the WS treatment became more pronounced such that ovary fresh weights in WS plants were lower than in WW plants along the length of the ear (Figure 4f), and collapsed ovaries were evident in the apical regions of the ear in WS plants (Figure 4a).

Silk elongation was strongly impacted by water deficit imposition in the basal regions of the ear in both experiments (Figure 5). At SE, silks along the entire length of the ears were shorter in the WS than the WW treatment in Exp I, but in Exp II silks in the apical third of the ears were similar in lengths in the two treatments. Silk lengths peaked at the 6th–10th rings (average 22.9 cm) in Exp I and the 4th–10th ring (>16 cm) in Exp II in WW plants, while peak silk lengths in WS plants were observed at the 11th–15th rings (average 12.9 cm) in Exp I and the 6th–13th ring (>12 cm) in Exp II (Figure 5a,b). Although apical silks in WW and WS treatments were of the same lengths at SE in Exp II, the WS treatment was associated with limited silk extrusion from the husks at SE+6d in the apical and basal regions of the ears in both experiments (Figure 5c,d).

### 3.5 WS increased sugar concentrations in ovaries and silks prior to and on the first day of silk emergence

**FIGURE 6:**
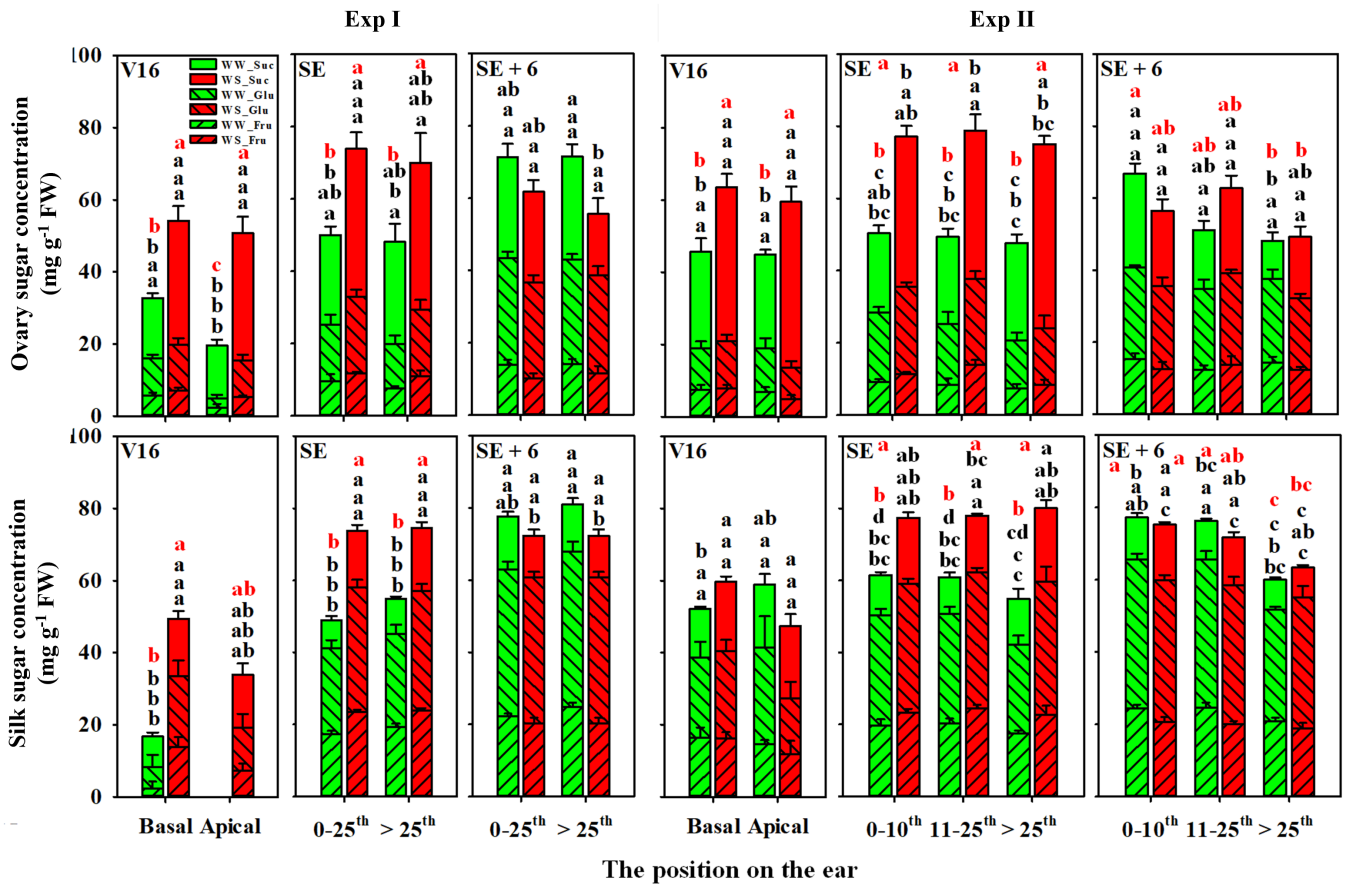
The concentrations (mg g^−1^ FW) of fructose (Fru, left slash filled), glucose (Glu, right slash filled), and sucrose (Suc, blank filled) in ovaries and silks from basal and apical regions of the ear at the 16‐leaf stage (V16) in Experiment I (Exp I) and Experiment II (Exp II), in basal (0–25th rings) and apical (>25th rings) ovaries and silks at the 1st of silk emergence (SE) and 6 days after silk emergence (SE+6) in Exp I, and in basal (0–10th rings), middle (11–25th rings), and apical (>25th rings) ovaries and silks at SE and SE+6 in Exp II under well‐watered (WW, green) and water‐stressed (WS, red) conditions. Error bars stand for standard errors. Different letters mean significant differences in the concentrations of the individual (black letters) or total (red letters) sugar among all ovaries or silks at the *p* < .05 level.

The concentrations of fructose, glucose, and sucrose in developing ovaries and silks were measured at V16, on the first day of silk emergence (SE), and at SE+6d (Figure 6). Total soluble sugar concentrations in WW ovaries and silks generally increased from V16 to SE+6d, more noticeably in Exp I than Exp II. However, in WS treatments, total soluble sugar concentrations in both tissues peaked at SE. WS increased sucrose concentrations in ovaries of both basal and apical ear sections at V16 in both experiments, and also the concentrations of fructose and glucose in the apical section in Exp I. At SE, water deficit increased the sucrose concentration in basal (0–25th) ovaries in Exp I, and in the basal (0–10th), middle (11–25th), and apical (>25th) ovaries in Exp II. However, fructose and glucose concentrations were not responsive to water treatment in Exp I, and only increased in WS compared to WW treatments in the middle ovaries in Exp II. At SE+6d, the concentrations of all soluble sugars individually or combined did not differ between WS and WW plants in either of the two experiments, except for a decrease in sucrose concentration in apical ovaries (>25th) in Exp I.

Similar to ovaries, the total sugar concentration in silk increased in response to water deficit at V16 and at SE, but was not different between WW and WS at SE+6d in Exp I. Further, the elevated total sugar concentration in WS compared to WW at V16 and SE was the result of greater concentrations of all the three measured soluble sugars. Although total sugar concentration did not differ between WW and WS at SE+6d, WS resulted in a lower fructose concentration in apical silks. In Exp II, total soluble sugar concentrations of silks were not influenced by water treatment at V16, but water deficit increased sucrose concentration in basal silks. However, just like in ovaries, total soluble sugar concentrations were greater in WS than in WW treatments at SE, but did not differ at SE+6d. The elevated concentrations in WS at SE were associated with increased sucrose concentration in basal silks (0–10th) and increased concentrations of all measured sugars in middle (11–25th) and apical (>25th) silks. The total sugar concentrations were comparable between WW and WS at SE+6d in all regions of the ear, even though water deficit decreased fructose in basal (0–10th) and middle (11–25th) silks and increased sucrose in basal (0–10th) silks.

The sugar contents (μg ovary^−1^) were calculated as the sugar amount per individual ovary at SE and SE+6d (Figure S3). Like sugar concentrations, total soluble sugar contents of ovaries increased from SE to SE+6d in all ovaries in WW conditions in both experiments. In basal ovaries of WW plants it more than doubled (2.2‐fold, +247 μg), and in apical ovaries it nearly quadrupled (3.8‐fold, +199 μg) from SE to SE+6d in Exp I. In WW plants of Exp II, total soluble sugar contents of basal ovaries (0 – 10th) increased by 208 μg per ovary (2.0‐fold), 131 μg (1.9‐fold) per middle ovary (11–25th), and 45 μg (2.3‐fold) per apical ovary (>25th) from SE to SE+6d. In the WS treatment of Exp I, total sugar content per ovary increased significantly from SE to SE+6d in basal ovaries (1.3‐fold, +59 μg), but not in apical ovaries. In the WS treatment of Exp II, sugar contents did not increase significantly between SE and SE+6d at any of the ovary positions.

Water treatment did not influence the ovary contents of fructose, glucose and sucrose at SE in Exp I. In contrast, at SE+6d, the total sugar content as well as the contents of each sugar in both basal (0–25th) and apical (>25th) ovaries were reduced by WS. In Exp II, water deficit increased sucrose content in basal (0–10th) ovaries and all sugars in middle (11–25th) ovaries at SE. At SE+6d, WS reduced contents of all sugars in basal (0–10th) ovaries, but did not affect sugar contents in middle and apical ovaries.

### 3.6 Correlation of individual ovary fresh weight with silk length

**FIGURE 7:**
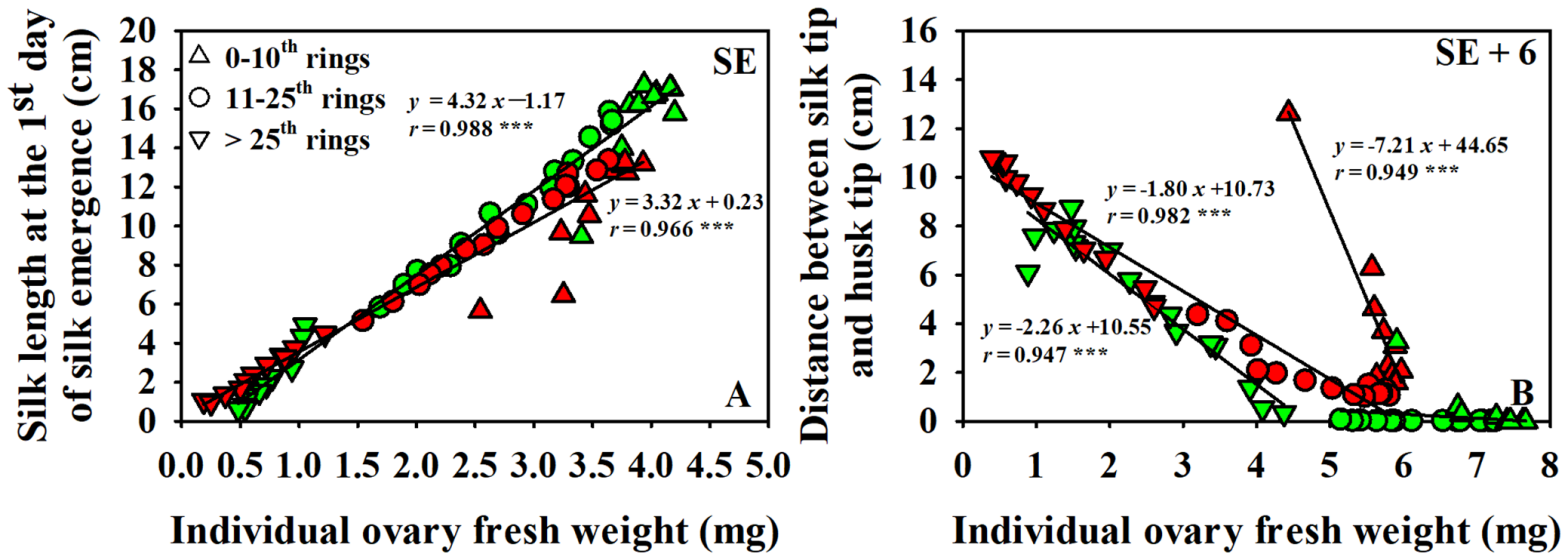
Correlation of individual ovary fresh weights with silk lengths at the 1st of silk emergence (SE, a) and the distances between silk tips to husk tips 6 days after silk emergence (SE+6, b) in both well‐watered (WW, green) and water‐stressed (WS, red) treatments. The linear regressions were fitted by pooling data from all positions of each treatment together at SE, while linear regression was fitted for ovaries above the 25th ring in WW treatment but separately for ovaries below and above the 10th ring in WS treatment at SE+6d. The corresponding equations with *r*‐value showed in the panels. The normal triangles represent data from ovaries located at 0–10th rings, circles for data from ovaries at the 11–25th rings, and inverted triangles for data from ovaries above the 25th rings in both WW and WS treatments. *** represents the correlation significant at *p* < .001 level.

The relationships between individual ovary fresh weights and silk lengths at SE and at SE+6d were explored using data from Exp II. Strong linear relationships were observed between ovary fresh weight and silk length at SE for both WW and WS treatments (Figure 7a). The regression lines for the two water treatments intersected at an ovary fresh weight of 1.4 mg. This point also separated ovaries below and above the 25th rings in both treatments. At equal mass, ovaries below the 25th ring supported longer silks in WW than in WS plants, whereas ovaries above the 25th ring exhibited longer silks in the WS than the WW plants. The relationship between individual ovary fresh weights and distances from silk tips to husk tips was more complicated (Figure 7b). Specifically, a strong negative correlation existed between these two indicators for ovaries above the 25th rings in WW plants with an x‐axis intercept of 4.67 mg. All ovaries with weights above 4.67 mg in 0–25th rings had extruded silks and were excluded from the regression analysis. In contrast, in WS plants, not all silks from the basal positions were fully extruded such that two regression lines were fitted in WS plants, one for rings ≥11 and one for the basal 10 rings. The slopes of the lines fitting these two groups indicate that the distances between the silk tips and husk tips changed more dramatically per unit of ovary weight for basal than for middle and apical ovaries (≥11th). The *x*‐axis intercepts of these two lines in WS treatment were 5.96 and 6.19 mg, respectively.

## 4 DISCUSSION

For the current study, we exposed B73 maize inbred lines to water deficit stress during silk elongation in two independent greenhouse experiments. The pot size and potting media used for these experiments were selected to achieve a compromise between a very rapid and a slow imposition of water deficit stress, with the goal of closely matching the target WS level with the period of silk elongation. As shown in Figure 1, the dry‐down was imposed over the course of 5 days in both experiments and induced significant differences in pre‐dawn and mid‐day leaf water potentials. It is important to note that the stress level as indicated by mid‐day leaf water potentials, was somewhat less severe during Exp I than during Exp II. This difference in stress level, likely caused by differences in day lengths and vapor pressure deficits associated with the time of the year during which the two expeirments were conducted (spring vs. late summer), modulated the relative extent of some of the responses between the two experiments. Nevertheless, the impact of water defcit stress on the measured traits followed the same overall pattern in both experiments.

### 4.1 Silk extrusion failure was the greatest contributor to kernel loss in response to water deficit stress imposition during early silk elongation

In maize, successful development of ovaries into fully mature kernels can be disrupted in various ways, including as a result of incomplete ovary differentiation (Otegui & Andrade, 2000), late‐appearing silks or silk extrusion failure (Cárcova & Otegui, 2001; Fuad‐Hassan et al., 2008; Oury, Tardieu, et al., 2016; Rossini et al., 2020), and failure of the fertilized ovary during initial kernel development (Westgate & Boyer, 1986). Even in the absence of water deficit, disruptions of developmental events can occur, and they are rarely limited to a single reason; rather, the primary reason for unsuccessful kernel development and filling largely depends on the environmental conditions at critical stages of development (Borrás & Vitantonio‐Mazzini, 2018; Messina et al., 2019; Turc & Tardieu, 2018). For example, decreased endosperm cell division affects kernel establishment under post‐pollination water deficit (Ober et al., 1991; Setter et al., 2001), while kernel abortion triggered by carbon limitation after fertilization has been documented as the main reason for kernel loss when WS occurs within 6 days after silk emergence (McLaughlin & Boyer, 2004b; Zinselmeier et al., 1999). Further, ovaries with silks that were not extruded 2 days before silk growth cessation largely aborted and accounted for more than 90% of kernel losses induced by a 2‐week post‐tasseling water deficit stress (Oury, Tardieu, et al., 2016). In this study, the WS treatment was started at silk initiation at about the 14‐leaf stage and plants were re‐watered 3 days after silking, resulting in water deficit stress in earlier developmental stages than in the aforementioned studies. This early imposition of WS caused a 58% reduction in kernel number (average across two experiments), with 68% of this kernel number reduction that can be attributable to failed silk extrusion. WS decreased silk elongation and altered the pattern of silk appearance which caused this large percentage of non‐emerged silks (Figures 2 and 5).

Silk appearance can proceed over the course of a week or more under WW conditions, but can be suspended early (e.g., 2–3 days after silk emergence) in response to water deficit (Messina et al., 2019; Oury, Tardieu, et al., 2016). DeBruin et al. (2018) reported that silk emergence occurred in two phases consisting of an initial surge in the first 1–2 days, and resulted in the extrusion of >70% of silks, followed by a slower rate of emergence over the course of several days. Plants in this study also exhibited this pattern, that is, silk emergence was intensive early (within the first 3–4 days) after initial silk appearance in both WW and WS plants, followed by reduced daily silk emergence and ultimately shortened total duration of silk emergence in the WS treatment (Figure 2). As such, even though early pollinated ovaries can exhibit primigenic dominance and negatively affect the development of late‐pollinated ovaries (Cárcova & Otegui, 2001, 2007; Shen et al., 2018; Turc & Tardieu, 2018), it is unlikely that kernel abortion caused by such temporal effects was the main cause of kernel losses in our study because about 81% silks (average across two experiments) in WW plants and 86% silks in WS plants extruded within 4 days after the first silks emerged (Figure 2). Rather, the combined effect of constrained silk elongation and silk growth cessation within the husks in the WS plants reduced emerged silk percentages (40% of the total floret number in WS compared to 70% in WW plants) (Figures 2, 3 and 5), and shortened total duration of silk extrusion (average of 4.9 d for WS vs 7.5 d for WW in Exp I and 7.3 d vs 8.7 d in Exp II) contributed to the 68% reduction of final kernel number (Figure 2).

### 4.2 Silk elongation was limited by low water potential and associated with slow ovary growth rates under water deficit stress conditions

Turc et al. (2016) showed that the response pattern of silk growth is similar to those of leaves in that they closely follow hydraulic cues including soil water status and evaporative demand, indicating a common hydraulic control partly shared among vegetative and reproductive organs for their expansive growth relative to changes in environmental conditions. However, the sensitivity of silk elongation to water deficit is greater than that of other tissues because of comparatively weak osmotic adjustment and associated susceptibility to turgor loss (Westgate & Boyer, 1985). Indeed, silk elongation is very sensitive to water potential, declining rapidly below about −0.4 MPa and reaching a threshold for growth at about −0.75 MPa, while the threshold is about −1.0 MPa for leaves and − 1.4 MPa for nodal roots (Bassetti & Westgate, 1993a; Westgate & Boyer, 1985). In this study, water potentials of both apical and basal silks at the time the first silk emerged (SE) were − 0.92 MPa (apical) to −0.89 MPa (basal) in WS plants and − 0.56 (basal) to −0.55 MPa (apical) in WW plants (Figure S4). Thus, silk water potentials of WS plants were below the previously reported threshold for silk elongation, which is consistent with the shorter silks and growth cessation of some silks that never protruded the husk in the WS compared to the WW treatment (Figure 5). Most of the arrested silks originated from ovaries that were collapsed (Figure 4), but the timing and sequence of events of silk growth arrest and ovary collapse were not investigated. The collapsed ovaries, evidence of premature growth cessation, might be triggered by enhanced programmed cell death (PCD) since the transcript abundance of negative regulators of PCD decreased and some positive regulators increased in drought‐stressed ovaries (Kakumanu et al., 2012). However, further studies are needed to ascertain the mechanisms underlying the collapse of ovaries and their link with silk elongation.

Longer silks generally were associated with ovaries with greater fresh weights and were less prone to fail to extrude from the husks; however, some exceptions were observed, especially when comparing WW and WS treatments (Figure 7). Silks at rings 5–25 with average ovary fresh weights ranging from 5.2 to 7.7 mg were all extruded by SE+6 in WW plants. In contrast, in WS plants an average of 22% of silks from rings 2 – 18 did not emerge by SE+6, even though average ovary fresh weights of rings 2–18 ranged from 5.3 to 6.0 mg (Figures 3, 4 and 7). Instead of ovary fresh weight per se, average ovary growth rates (calculated for the period from SE to SE+6) may be more informative relative to silk extrusion. Indeed, average growth rates of ovaries from positions with fully exposed silks were 0.53–0.65 mg day^−1^ in WW plants, while those of ovaries from positions with non‐fully exposed silks ranged between 0.15 and 0.57 mg day^−1^ in WW plants. In WS plants, the maximum ovary growth rate at any position was only 0.47 mg day^−1^, and not a single ring of ovaries exhibited successful silk extrusion from all ovaries. Analysis of data from both WW and WS plants revealed a close correlation between average distances from silk tips to husk tips and average ovary growth rates (*r* = .866, *p* < .0001). Thus, similar to Borrás et al. (2007), ovary growth rates may serve as indicator for the evaluation of silk lengths and silk extrusion irrespective of water treatments, but, the mechanistic interrelationships between ovary growth and silk elongation remain to be elucidated. The results presented here are consistent with the strong relationship of ovary growth rate with maize kernel set that has been reported by Cárcova and Otegui (2007). Similarly, greater ear tip‐to‐base ratio in ovary growth rates were observed in WW (0.56) compared to WS (0.33) and were associated greater kernel numbers in WW than WS treatments (Figures 3 and 4) in this study.

### 4.3 The spatial distribution of kernel loss was associated with reduced silk emergence from husks and ovary losses despite silk emergence and greater sugar concentrations

In this study, kernel loss occurred along the whole length of the ear in WW and WS treatments but was more pronounced in WS treatments and also at the apical and basal ends of the ear (Figure 3). Silk initiation first occurs at ovaries in the lower middle region of the ear, usually at floret positions of rings 5–15, and then gradually proceeds toward the basal and apical ends of the ear. WS does not change this temporal pattern of silk initiation but can affect the growth and sequential emergence of silks (Bassetti & Westgate, 1993b; Cárcova et al., 2003; Turc et al., 2016). Here, water deficit with onset at silk initiation reduced silk lengths throughout the entire ear and, not surprisingly, to a greater extent for later developing silks (Figure 5). Silk growth arrest inside the husks resulted in ovary failure due to the lack of fertilization and was the primary source (68%) of kernel loss in this study. The remaining 32% of kernel loss stemmed from ovaries that failed to develop despite having emerged silks. Since plants were hand‐pollinated on a daily basis, these ovary failures could not be attributed to a lack of pollination. Rather, developmental failure within ovaries and/or post‐pollination kernel abortion were likely the primary reasons underlying the observed remaining kernel loss. Indeed, Bassetti and Westgate (1993a) previoulsy found that kernel loss was primarily caused by a developmental failure within ovaries instead of the loss of receptivity of silks when WS was imposed within 4 days after silking. This also is consistent with findings by Otegui et al. (1995) that artificial pollination of late‐emerged silks with fresh pollen grains did not result in more kernels per ear, indicating that the function of ovaries with late‐emerging silks was impaired by WS. Primigenic dominance effects on kernel abortion have been documented and may have played a role in the observed pattern of kernel loss (Cárcova et al., 2000; Cárcova & Otegui, 2001; Shen et al., 2018; Uribelarrea et al., 2008). Shen et al. (2018) proposed that apical abortion might be related to weak competitiveness for maternal assimilates and the inhibition of sucrose utilization because of delayed pollination‐induced invertase reduction.

Water deficit stress alters carbon supply and metabolism and can cause carbon starvation of ovaries when it occurs around pollination (McLaughlin & Boyer, 2004a, 2004b; Zinselmeier et al., 1999). Interestingly, somewhat earlier WS during silk emergence until 2 days after pollination can result in increased ovary sugar concentrations but nonetheless cause kernel loss, which may be related to suppressed flow of assimilate into the ear due to a failure of sugar utilization (Schussler & Westgate, 1995). Andersen et al. (2002), suggested that abundant carbohydrates in ovaries under WS may be related to an early response of soluble invertase to WS, which could affect sucrose delivery and use during the abortion sensitive phase. Consistent with those studies, WS increased or maintained the sugar concentration of developing ovaries and silks at V16 and silk emergence in the present work, but, remarkably, either did not significantly change sugar concentration or decrease it in ovaries 6 days after silk emergence (Figure 6). This increased sugar concentrations under drought may in part be attributable to the lower relative sensitivity of photosynthesis to water deficit as compared to growth (Boyer, 1970; Hummel et al., 2010; McDowell, 2011; Pantin et al., 2013). Also, higher sugar concentrations may play a role in coping with water deficit stress through osmotic adjustment and in ROS scavenging (Henry et al., 2015; Pantin et al., 2013; Saddhe et al., 2020).

Water deficit stress clearly impacted both photosynthesis and ovary growth in this study (Figure S1 and Figure 4). Ovary fresh weights were significantly greater in WW than WS treatments (Figure 4), and sugar contents increased 1.9‐ to 3.8‐fold across all ovaries of WW plants from SE to SE+6, but only increased 1.1‐ to 1.3‐fold for ovaries of WS plants in Exp I and 0.8‐ to 1.2‐fold in Exp II (Figure S3). Thus, it appears that WS strongly inhibited assimilate flux into the developing ovaries during SE to SE+6, possibly caused by a direct effect of water deficit stress on ovary expansion or the ability to utilize available carbohydrates, maybe due to reduced invertase activity, and ultimately limited sink strength (Figures 4, 6, 7). However, whether, and if so how, reduced sink strength or the rate of carbohydrate transport into developing ovaries may be related to the 32% kernel loss at positions with emerged silks remains to be elucidated. That said, hormonal signaling likely plays an important role in regulating processes leading to kernel losses (Turc & Tardieu, 2018) and, given the discrepancy of responsiveness to plant hormones like ethylene (Cheng & Lur, 1996; Shen et al., 2019) and abscisic acid (Ober et al., 1991; Ober & Setter, 1990) between basal and apical ovaries, may critically influence the spatial pattern of kernel abortion observed along the ear.

## 5 CONCLUSION

In this study, water deficit stress imposed early during silk initiation (14 leaf stage) through 3 days after silk emergence, resulted in a 58% kernel number reduction with 68% of this reduction attributable to arrested silks within the husks and the remaining 32% to ovary failures. The non‐emerged silks and failed ovaries followed similar patterns of spatial distribution along the length of the ear under WW and water deficit stress conditions, with the lowest percentages of failures in the middle region and gradually increased failure percentages toward the apical and basal regions of the ear. Water deficit reduced silk elongation largely dependent on their position, and the severity of impact was inverse to the temporal pattern of silk initiation. Silk water potentials below the threshold for silk elongation resulted in shorter silks, which also were associated with ovaries that exhibited slower growth rates. While photosynthesis was strongly reduced under water deficit conditions, ovary and silk carbohydrate concentrations were either significantly increased (early) or maintained (SE+6) relative to WW conditions. Increased sugar concentrations in ovaries of water stressed plants may in part be the result of reduced ovary expansion and limited ability to utilize available carbohydrates, and a reduction in carbohydrate supply to reproductive tissues also might be a reflection of dysfunction of carbohydrate utilization in ovaries. Overall, although arrested silks within husks were the primary reason for kernel loss due to water deficit stress early during silking, about one third of observed kernel losses were due to other reasons and were associated with ovaries with reduced growth rates despite high sugar concentrations. Future studies should examine the impact of the rate of stress imposition on silk elongation and carbohydrate dynamics and their relative importance, especially under field conditions where light intensities exceeding those in greenhouses may enhance source strength and differences in plant available soil water in different soil types can impact the rate of stress imposition and thus the extent of acclimation that may occur over the course of the dry‐down period.

## CONFLICT OF INTEREST STATEMENT

No conflict of interest was declared by the authors.

## Supporting information


Data S1.

Figure S1.

Figure S2.

Figure S3.

Figure S4.

Data S2.

## Data Availability

Data are provided as supplementary material.
