# Supplementary figures and images for: Pre‐silking water deficit in maize induced kernel loss through impaired silk growth and ovary carbohydrate dynamics
Source: Plant Environ Interact. 2024 Apr 6;5(2):e10141. doi: 10.1002/pei3.10141 (PMC10998497; doi:10.1002/pei3.10141)

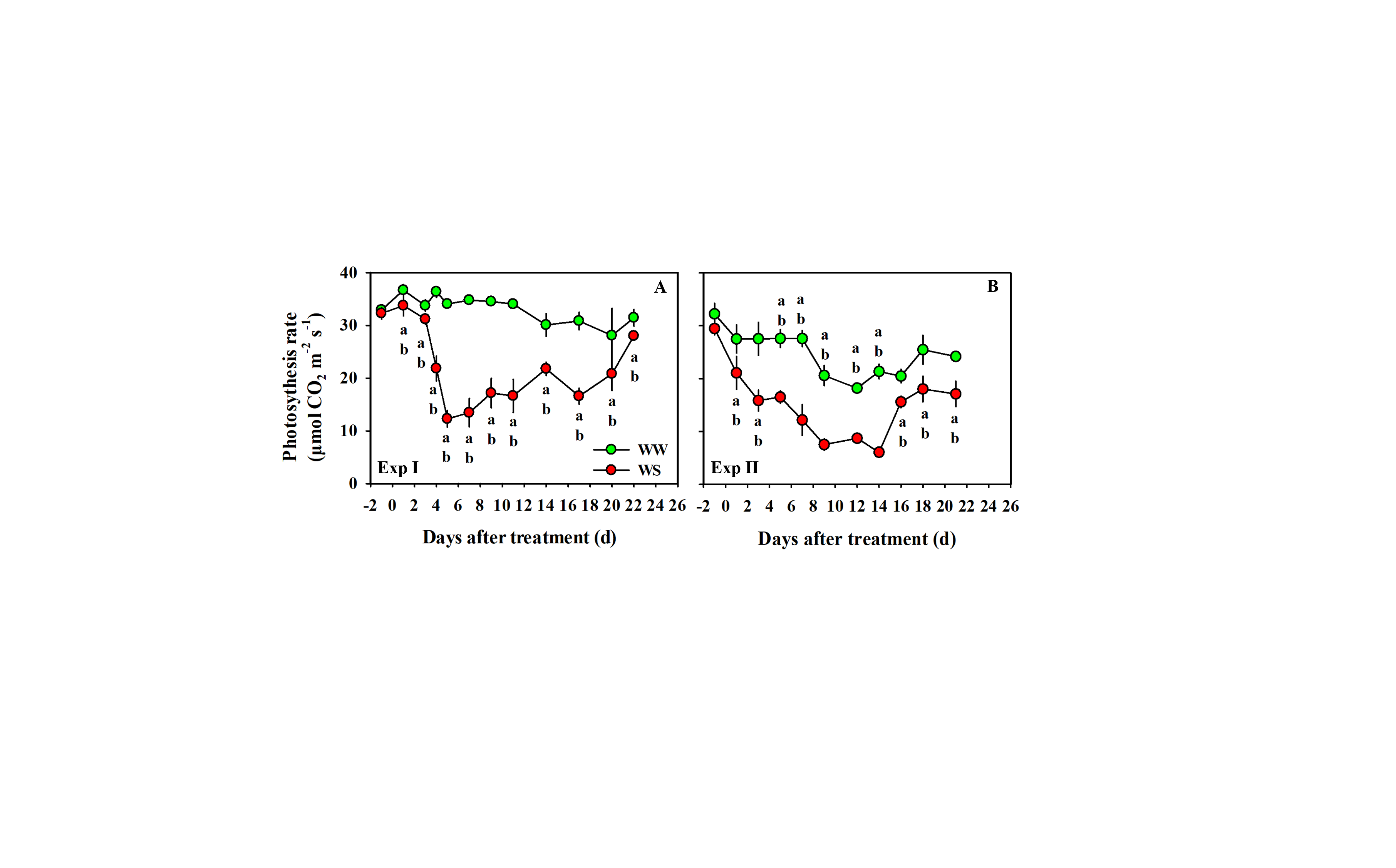

Supplement: Supplementary file 2 — Figure S1. [file PEI3-5-e10141-s005.tif]

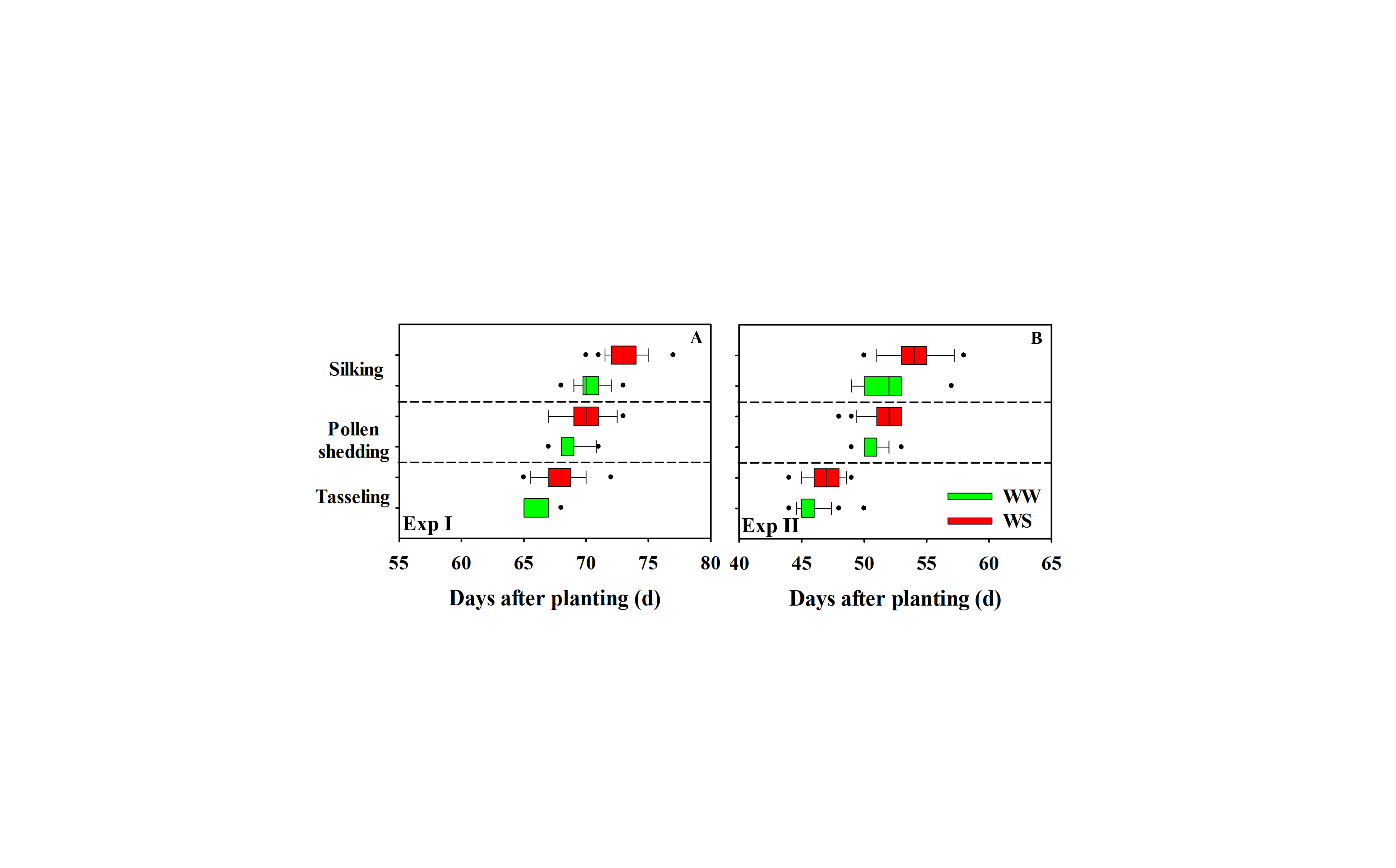

Supplement: Supplementary file 3 — Figure S2. [file PEI3-5-e10141-s006.tif]

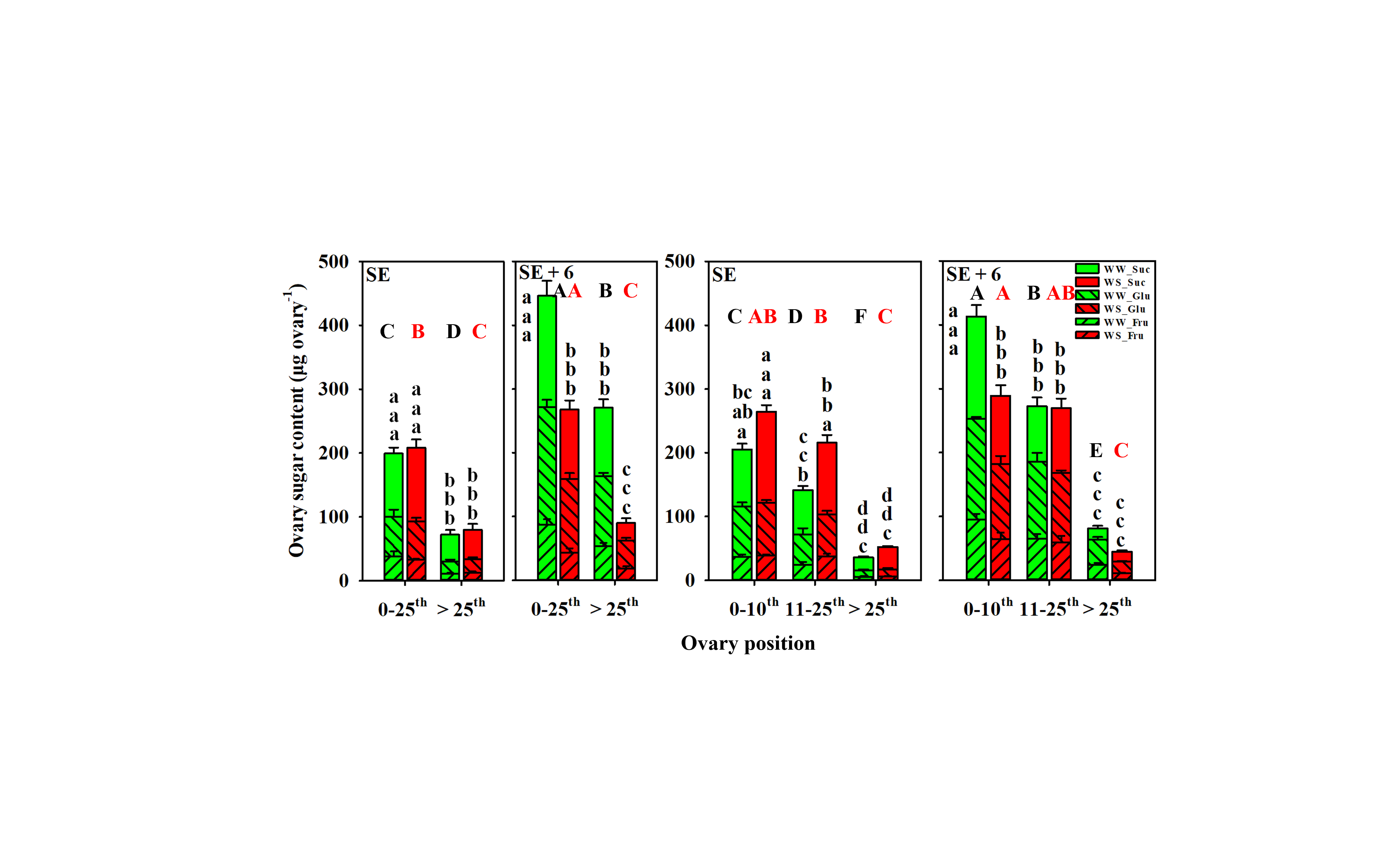

Supplement: Supplementary file 4 — Figure S3. [file PEI3-5-e10141-s002.tif]

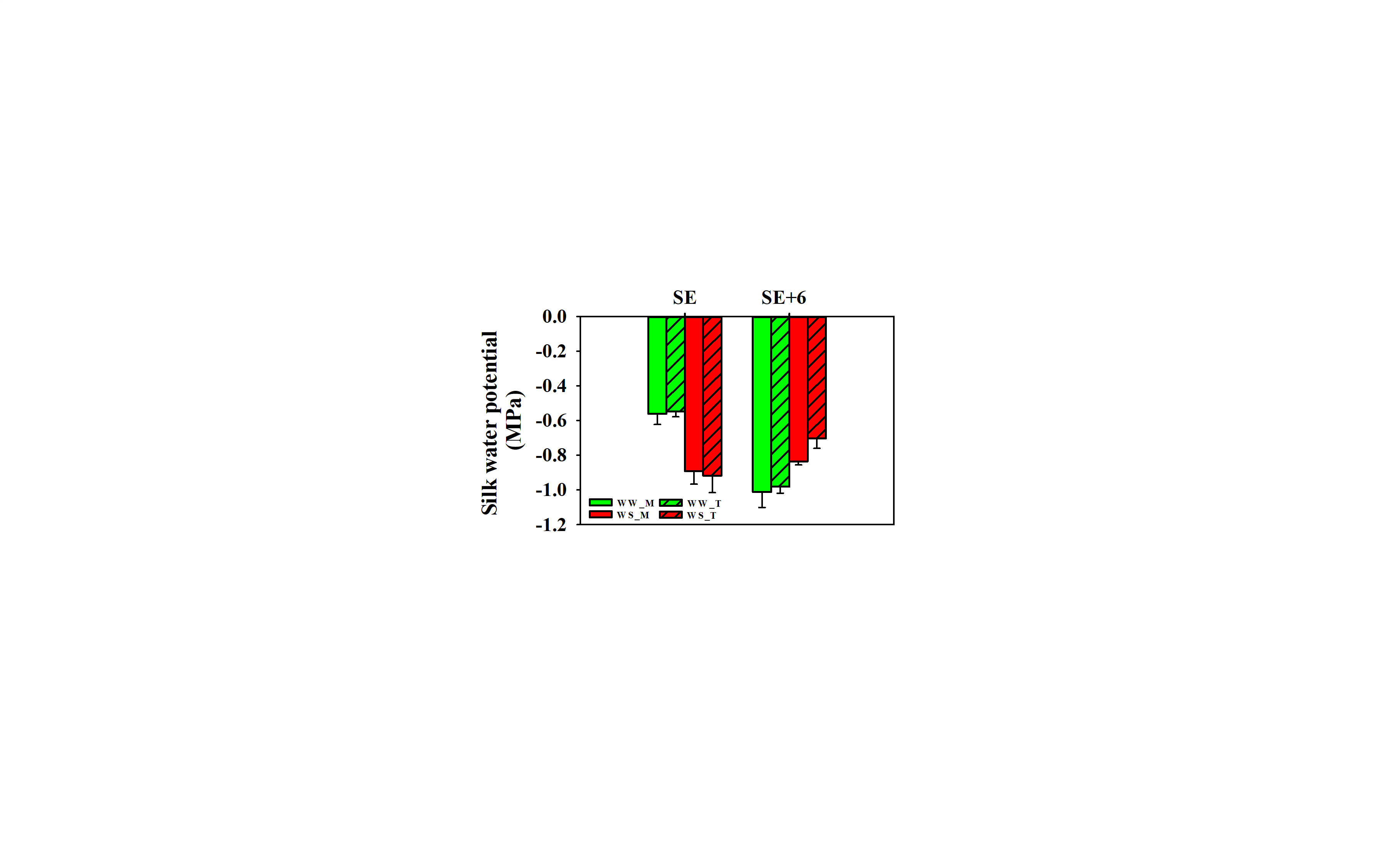

Supplement: Supplementary file 5 — Figure S4. [file PEI3-5-e10141-s001.tif]
