# Supplementary material for: Pre‐silking water deficit in maize induced kernel loss through impaired silk growth and ovary carbohydrate dynamics
Source: Plant Environ Interact. 2024 Apr 6;5(2):e10141. doi: 10.1002/pei3.10141 (PMC10998497; doi:10.1002/pei3.10141)
Supplement: Supplementary file 6 — Data S2. [file PEI3-5-e10141-s003.docx]

**Supplementary data**

**Fig. S1.** The dynamic of photosynthesis rates (*P*_n_, μmol CO_2_ m^-2^ s^-1^) of well-watered (green, WW) and water-stressed (red, WS) plants in Experiment I (Exp I, A) and Experiment II (Exp II, B). Different letters mean significant differences between treatments at the *p*<0.05 level, and error bars stand for standard errors.

**Fig. S2.** The timing to tasseling, pollen shedding, and silking of well-watered (green, WW) and water-stressed (red, WS) plants in Experiment I (Exp I, A) and Experiment II (Exp II, B). Different letters mean significant differences between treatments at the *p*<0.05 level.

**Fig. S3.** The contents (μg ovary^-1^) of fructose (Fru, left slash filled), glucose (Glu, right slash filled), and sucrose (Suc, blank filled) in basal (0–25^th^ rings) and apical (>25^th^ rings) ovaries at the 1^st^ of silk emergence (SE) and 6 days after silk emergence (SE+6) in Experiment I (leaf two panels), and basal (0–10^th^ rings), middle (11–25^th^ rings), and apical (>25^th^ rings) ovaries at SE and SE+6 in Experiment II (right two panels) under well-watered (WW, green) and water-stressed (WS, red) conditions. Different letters mean significant differences among all ovaries at the same stage (small letters) or among ovaries from the same treatment but sampled at different timepoints (capital letters) at the *p*<0.05 level.

**Fig. S4.** Water potentials of basal (blank filled bars) silks from well-watered plants (WW_M) and water-stressed plants (WS_M) and apical (slash filled bars) silks from well-watered plants (WW_T) and water-stressed plants (WS_T) at the 1^st^ of silk emergence (SE) and 6 days after silk emergence (SE+6) in Experiment I.
